# Disruption of microbial cell morphology by *Buxus macowanii*

**DOI:** 10.1186/s12906-020-03049-5

**Published:** 2020-08-31

**Authors:** B. Ngobeni, S. S. Mashele, N. J. Malebo, E. van der Watt, I. T. Manduna

**Affiliations:** 1 Department of Health Sciences, Central University of Technology, Bloemfontein, South Africa; 2 Department of Life Sciences, Central University of Technology, Bloemfontein, South Africa; 3 Department of Soil, Crop and Climate Sciences, University of the Free State, Bloemfontein, South Africa; 4 Centre for Applied Food Sustainability and Biotechnology (CAFSaB), Central University of Technology, Private Bag X20539, Bloemfontein, 9300 South Africa

**Keywords:** Antimicrobial activity, *Buxus macowanii*, Neophytadiene

## Abstract

**Background:**

Microbial infections are one of the major causes of death globally. This is attributed to the rising costs of primary healthcare and its inaccessibility especially in developing countries. Moreover, there has been an increase in microbial strains that have reduced susceptibility to antimicrobial drugs. Research on the antimicrobial properties of medicinal plants, which could address these problems, has become more important as they present fewer side effects when compared to the antibiotics currently in use. This study evaluated the antimicrobial properties of a methanolic extract from *Buxus macowanii* in order to assess its potential in the development of novel antimicrobial drugs.

**Methods:**

Antimicrobial activity of the extract was evaluated using the broth microdilution method. The effects of *B. macowanii* on the morphology of *B. cereus* were observed using Scanning and Transmission electron microscopy. Chemical profiling of the plant extract was performed using the GCMS.

**Results:**

The extract showed antimicrobial activity against all the microbial species used. Microscopic examination of the cells of *B. cereus* cells treated with *Buxus macowanii* showed some changes in morphology such as damage of the cell wall, swelling of the cells and incomplete cell division that eventually resulted in cell death. Neophytadiene, an antimicrobial compound was detected in the extract using GCMS.

**Conclusion:**

The morphological disruptions of the cell wall of *Bacillus cereus* explain the antimicrobial properties of *B. macowanii* and indicate its possible application in the development of natural antimicrobial drugs.

## Background

The use of western medicine has always been associated with a number of problems that make more challenging the treatment of infectious diseases. Mainstream antimicrobials are marred by complications such as limited accessibility for marginalised communities, high purchase prices and after-use side effects [1, 2]. Additionally, the emergence of drug resistant pathogens and an increase in opportunistic infections in people with Human immunodeficiency virus/Acquired Immune Deficiency Syndrome (HIV/AIDS) and those on chemotherapy also make the treatment of infectious diseases complicated and difficult [3, 4]. These problems have encouraged research into alternative sources of novel drugs and drug leads that could overcome such challenges.

Plants have been used by millions of people as a source of food and for medicinal purposes for thousands of years [2, 5]. An estimated 80% of the world’s population and 80% of black South Africans still rely on traditional medicine for their primary health care needs [6]. Traditional medicine has not only gained popularity due to its effectiveness against diseases but because sometimes it is the only therapy that is available in rural areas in the context of developing countries [7]. The use of traditional medicine in South Africa is further attributed to accessibility, affordability, fewer side effects and extensive knowledge and expertise of some people within these communities [8, 9]. Traditional knowledge and ethnobotany provide clues of medicinally effective plants that could lead to the discovery and manufacture of new drugs. Medicinal plants, especially those used by traditional healers are considered the best options for the production of novel drugs because they have an unmatched chemical diversity [10, 11]. They contain bioactive compounds such as alkaloids, terpenoids, tannins, flavonoids, peptides and phenolic- compounds that are known to have antimicrobial, antiviral, antifungal, anti-inflammatory, anthelmintic and antioxidant activity [12].

This study focuses on the antimicrobial effects of a methanol extract from *Buxus macowanii* Oliv*.* commonly known in South Africa as “Cape Box”, “Umgalagala” or *“*Igalagala” in isiXhosa [13]. It is a small-growing, evergreen plant from the Buxaceae family [14] and it grows in the Eastern Cape, Mpumalanga and Limpopo provinces of South Africa. The plant is used by traditional healers to treat wounds, pain, gout, malaria, rheumatism and skin disorders [13, 15–18]. The traditional uses of *Buxus macowanii* stated above motivate this study on antimicrobial effects of its extracts and its potential in the development of new plant-based antimicrobials.

## Methods

### The plant extracts

The twigs and leaves of *B. macowanii* were collected in Mpumalanga by the National Museum Bloemfontein using the correct collection permits for their herbarium and collection number RB 829 was allocated. The National Museum of Bloemfontein also conducted formal identification of the plant and further deposited it into their own herbarium. The required application with the government in respect of permits, clearances and collection of plants complying with South African legislation was lodged and granted. The plant material was washed, and oven dried at 40 °C for 72 h. The dried leaves and twigs were then combined, ground to a powder and extracted with 100% methanol. The methanol was evaporated at 40 °C under vacuum using a Buchi Rotavapor. The extract (No. 1544) was stored in a fridge − 20 °C until needed (Department of Soil, Crop and Climate Sciences, University of the Free State).

### Microbial cultures

Antimicrobial activity of the plant extract was evaluated using *Staphylococcus aureus* (ATCC 25923), *Clostridium perfringens* (ATCC 13126), *Pseudomonas aeruginosa* (ATCC 27853), *Enterococcus faecalis* (ATCC 29212), *Escherichia coli* (ATCC 25922), *Staphylococcus epidermidis* (ATCC 12228), *Bacillus cereus* (ATCC 13061) and the fungal species *Candida albicans* (ATCC 90028) and *Candida tropicalis* (ATCC 756). All the bacterial and fungal species were supplied by the National Health Laboratory Services, Bloemfontein, South Africa. All the microbial species were maintained in Mueller Hinton agar plates at temperatures of 4 °C. Prior to antimicrobial testing, the microorganisms were inoculated in Mueller Hinton broth and placed in a shaking incubator (100 rpm) for 24 h at 37 °C. Thereafter, a microbial suspension was prepared by diluting one millilitre of the culture in 100 ml of Mueller Hinton broth (1:100).

### Antimicrobial screening

Antibacterial and antifungal activity of the extract were evaluated using the broth microdilution method developed by Eloff [19]. The dried plant extract was dissolved in 5% dimethyl sulfoxide (DMSO). One hundred microliters of the bacterial suspension was pipetted into the 96 microwell plate already containing 100 μl of diluted plant extract to make a final volume of 200 μl in each well. The concentration of the plant extract ranged from 0.16 mg/ml to 2.5 mg/ml. The first control wells were filled with the culture medium only and the second control only contained the bacterial suspension. The third control was the solvent of extraction, methanol, at 5% [20, 21] and the plant extract alone was the fourth control. Chloramphenicol (0.125 mg/ml) [22] was used as a positive control in bacteria and Amphotericin B (0.03–1 μg/ml) [23] was used in fungi. The micro-well plates for bacteria were incubated for 24and 48 h for yeast. Thereafter, 40 μl of 4 mg/ml Iodonitrotetrazolium salt solution was added in each well. Microbial growth was indicated by a change of colour ranging from pink to violet after 10–30 min incubation. All samples were tested in triplicate. The MIC was defined as the lowest concentration at which no microbial growth was recorded.

### The microscopic analysis of antibacterial activity

The cell wall of *Bacillus cereus* (ATCC 13061) was examined using Scanning and Transmission Electron Microscopy after treatment with the methanol extract of Buxus macowanii. *B. cereus* was treated with different concentrations (0.2–2.5 mg/ml) of the methanol extract of *B. macowanii*. The untreated samples (control) and the treated samples were incubated at 37 °C for 24 h. After incubation, the cells were washed twice with 0.1 M phosphate buffer solution (PBS, pH 7.0) and were fixed using 3% glutardialdehyde and 1% osmiumtetroxide and kept for 2 h at − 4 °C. Thereafter, the cells were subjected to dehydration in ethanol at successive concentrations of 50, 70, 95, 2X100% followed by critical point drying using CO_2_ to remove ethanol. The samples were finally mounted on a specimen stub and coated with gold under vacuum followed by microscopic examination using SEM [4, 24]. For TEM, dehydrated bacterial cells were embedded by replacing acetone with epoxy to make slim sections suitable for microscopy examination. Samples were also further embedded using epoxy for 8 h at 70 °C in special moulds. The samples were cut into sections using an ultramicrotome and stained with 6% Uranyl and lead citrate followed by TEM examination [25, 26].

### Cytotoxicity evaluation of *B. macowanii*

The Sulforhodamine B (SRB) assay was carried out to evaluate the toxicity of the methanol extract of *B. macowanii* against WI-38 Normal human fetal lung fibroblast cells [27]. The WI-38 cells l from European Collection of Cell Culture (ECACC) were maintained at 37 °C, 5% CO_2_, 95% air and 100% relative humidity as a monolayer cell culture in EMEM supplemented with 10% fetal bovine serum (FBS), 2 mM L-glutamine and 50 μg/ml gentamicin. For cytotoxicity screening, the cells were inoculated in 96-well microtiter plates at a plating density of 10,000 cells/well and incubated for 24 h. After 24 h the cells were treated with the plant extracts at five different concentrations ranging from 6.25–100 μg/ml (5 x two-fold serial dilutions) and further incubated for 48 h. The untreated cells served as a control, the blank contained the medium without cells and etoposide was used as a standard at concentrations ranging from 100 μg/ml – 0.05 μg/ml (8 × 3-fold serial dilutions). After 48 h incubation, viable cells were fixed to the bottom of each well with cold 50% trichloroacetic acid, washed, dried and dyed with SRB. The protein-bound dye was removed with 10 mM Tris base and the optical density determination done at 540 nm using a multi-well spectrophotometer. The IC_50_ considered as 50% cell growth inhibition was determined by non-linear regression using GraphPad Prism 6.0. Cytotoxic activity was divided into 4 categories: Low Hazard (IC_50_ > 100 μg/ml), weak Hazard (30 μg/ml < IC_50_ < 100 μg/ml), moderate Hazard (5 μg/ml < IC_50_ < 30 μg/ml) and High Hazard (IC_50_ < 5 μg/ml). The Selectivity Index (SI) was expressed by dividing the IC_50_ with the MIC (SI = IC_5_MIC) [28].

### Gas chromatography mass spectrometry (GCMS)

The dried extract was dissolved in ethyl acetate, centrifuged and analysed by GCMS using an Agilent 6890 N linked to a Mass Detector 5975B. Total ion chromatograms (TIC) and their associated spectra were acquired in full scan mode using the Chemstation software linked to the GCMS. Peaks and their associated spectra were then searched using the Wiley 375 Mass Spectral Database. Due to the complexity of the chromatograms, there is a certain degree of spectral overlap. To overcome this, Automated Mass Spectral Deconvolution and Identification Software (AMDIS) was used to separate multiple overlapping spectra lying within single chromatographic peaks.

## Results

### Antimicrobial screening

The methanol extract from the twigs and leaves of *B. macowanii* showed antimicrobial activity against all the tested bacterial and fungal species. *B. macowanii* inhibited the growth of *Staphylococcus aureus, Staphylococcus epidermidis, Candida albicans, Candida tropicalis, Clostridium perfrengens and Pseudomonans aeruginosa* at the MIC of 2.5 mg/ml. *B. macowanii* also showed antimicrobial activity *against Enterococcus faecalis*, *Escherichia coli and Bacillus cereus* at an MIC of 1.2 mg/ml.

### Microscopic analysis of antibacterial activity

**Fig. 1:**
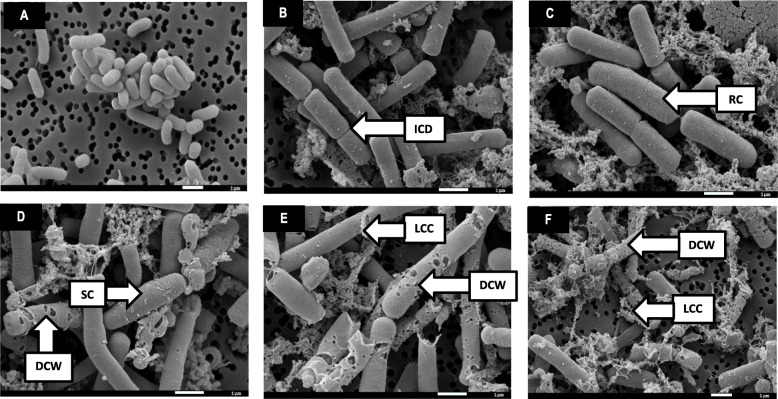
SEM photomicrograph of the concentration-dependent morphological changes exerted on *B. cereus* by the methanol extract of *Buxus macowanii*. **a** Control cells (untreated), (**b**) 0.156 mg/ml, (**c**) 0.31 mg/ml, (**d**) 0.625 mg/ml, (**e**) 1.25 mg/ml, (**f**) 2.5 mg/ml. Damaged cell wall (DCW) with the formation of holes on the cell surface; loss of cellular contents (LCC); incomplete cell division (ICD); Rough cell (RC) and swollen cells (SC)

The effects exerted by the methanol extract of *B. macowanii on* the cell shape and surface, the cell wall, the cytoplasmic membrane as well as on cell division and cell viability of *Bacillus cereus* are presented in Table 1. *B. cereus c*ells treated with *B. macowanii* showed concentration-dependent morphological changes that increased in severity as the concentration of the increased (Fig. 1). Under normal conditions the cells of *B. cereus* under the Scanning Electron Microscopy (SEM) were uniformly rod-shaped and had smooth cell surfaces (Table 1 and Fig. 1a).

**Table 1:** Morphological effects of the methanol extract from the of leaves and twigs of *B. macowanii* on *B. cereus*

Morphological changes	SEM		TEM	
	Control	***Buxus macowanii***	Control	***Buxus macowanii***
**Cell shape**	Uniformly rod-shaped	swollen cells (CWS)	Uniformly rod-shaped	Cells are swollen and contracted
**Cell surface**	Smooth cell surfaces	rough cell surface (RC)	Smooth cells surfaces	Rough cell surface (RC)
**Cytoplasmic membrane**	Intact	^a^Membrane damage (DWC) and loss of intracellular contents (LCC)	Cytoplasm was homogenously electron dense	Partial loss of cytoplasmic electron density
**Cell wall**	Intact	Damaged cell wall (DWC) (Perforations)	Intact	Cell wall distortion
**Cell division**	Complete	Incomplete cell division (ICD)	Complete	Incomplete cell division (ICD)
**Cell viability**	Viable	Cell death	Viable	Cell death

^a^No DNA extravasion carried out. However, the combination of the damage to the cell wall, perforations and other changes observed point to cytoplasmic membrane damage and loss of intracellular contents

**Fig. 2:**
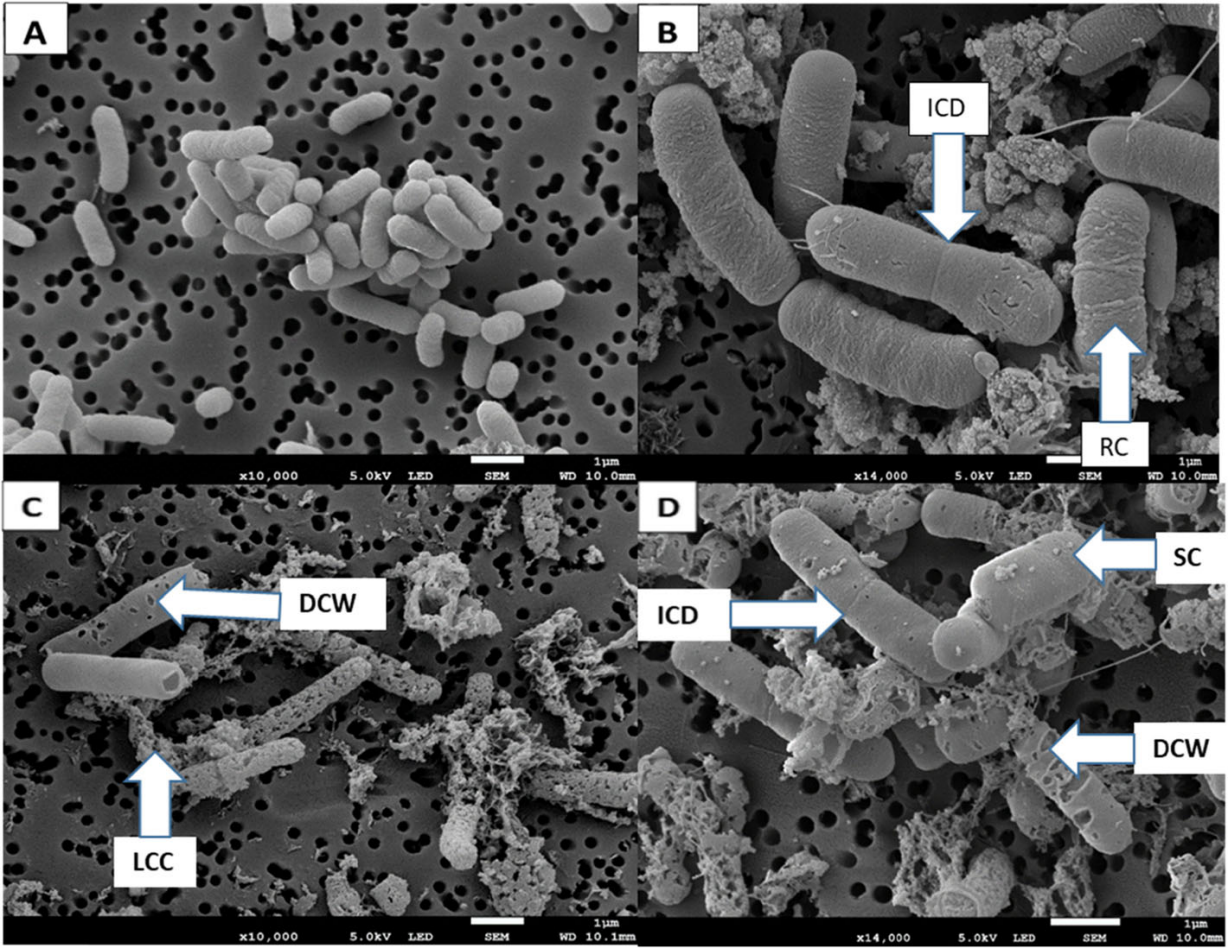
SEM Photomicrograph of the effects of *B. macowanii* at 2.5 mg/ml on the bacterial cell wall of *B. cereus*. **a** Control cells (untreated), (**b**) Positive control treated cells (Chloramphenical) and (**c** and **d**) different effects of *B. macowanii* on the bacterial cell wall and structure. Damaged cell wall (DWC) with the formation of holes on the cell surface; loss of cellular contents (LLC); incomplete cell division (ICD); rough cell (RC) and swollen cell (SC)

**Fig. 3:**
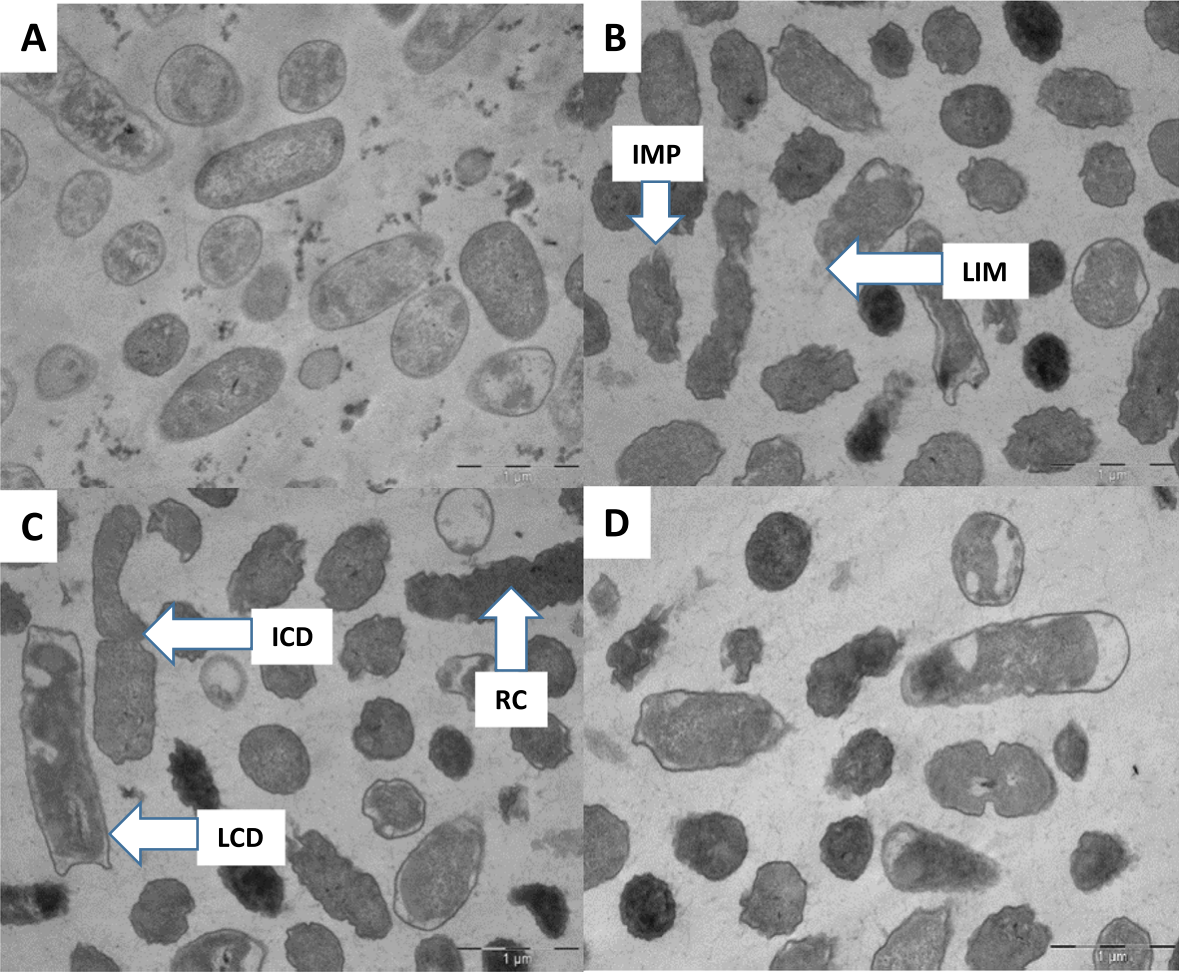
TEM Photomicrograph of the effects of *B. macowanii* at 2.5 mg/ml on the bacterial cell wall of *B. cereus*. **a** Control cells (untreated), **d** Positive control treated cells and (**b** and **c**) morphological changes of *B. cereus* after treatment with *B. macowanii*. Increased membrane permeability (IMP) that resulted in shrinkage of the cell; loss of intracellular material (LIM); incomplete cell division (ICD); roughness of the cell (RC) and loss of cytoplasmic density (LCD)

The Transmission Electron Microscopy (TEM) micrographs also showed that the cytoplasmic membrane and cell wall were intact under normal conditions (Fig. 3a). The SEM images (Fig. 2c and d) depicted structural changes such as damage on the cell wall (DCW), completely worn out cell walls and cytoplasmic membrane. The extract also caused incomplete cell division (ICD), swelling of the cell (CWS), cytoplasmic membrane damage and loss of intracellular contents (Table 1). Cells treated with the positive control (Chloraphenicol) at 0.025 mg/ml also showed bacterial structural changes similar to those seen in cells treated with *B. macowanii* such as roughness of the cell (RC), swollen cells (SC) and incomplete cell division (ICD) (Fig. 2b). *B. cereus* cells treated with *B. macowanii* at 2.5 mg/ml showed major structural changes on the cell wall that led to cell death in SEM (Figs. 1f and 2). The effects of *B. macowani*i on the morphology of the bacterial cells examined using TEM also confirmed the damaging effects of the plant extract on the bacterial cell structure (Table 1). The treated cells showed cell wall distortion (Fig. 3b). loss of intracellular or cytoplasmic contents, incomplete cell division, rough cell surfaces and separation of the cytoplasmic membrane from the cell wall when treated with *B. macowanii*. Additionally, the cells also showed partial to complete loss of electron density of both their cell wall and cytoplasm (LCD).

The SRB assay showed that the methanol extract of *B. macowanii* with an IC_50_ of 20.4 μg/ml was moderately hazardous (5 μg/ml < IC_50_ < 30 μg/ml) against the WI-38 cell line while etoposide was highly hazardous with an IC_50_ of 5.1. μg/ml. The Selectivity Index was 0.017.

**Fig. 4:**
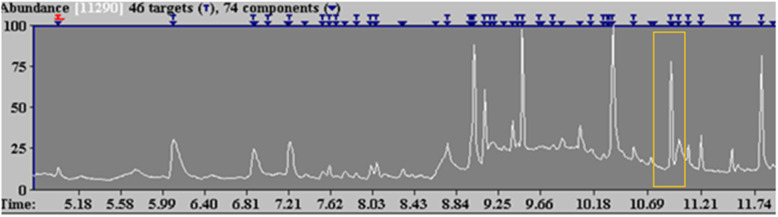
GCMS Total ion chromatogram of the methanol extract of *B. Macowanii*

GCMS analysis of the plant extract was conducted using the National Institution of Standard Technology (NIST) software and the Wiley Online Library. The identification of the compounds was based on the molecular weight, peak area and molecular formula, and only prominent peaks were selected for identification. A compound was only regarded as positively identified when the percentage of similarities was more than 90%. Neophytadiene from *B. macowanii* was detected at a retention time of 10.92 (Fig. 4).

## Discussion

The antimicrobial activity of *B. macowanii* against microorganisms such as *Candida spp, S. aureus, E. coli* is noteworthy because such microorganisms are responsible for most opportunistic infections and therefore have a negative impact on the health of the citizens of South Africa and the world at large [29]. Chloramphenicol was used as a positive control in our study and it is known to be effective against both the gram-negative and gram-positive bacteria [30]. The methanol extract of *B. macowanii* was effective against gram-negative and gram-positive bacteria used in our study. The inhibition of gram-negative, gram-positive bacteria and *Candida* is therefore of critical importance because of the challenges such as nosocomial infections, spread of multi-drug resistant strains, increase in mortality rate, frequent relapse and treatment failures that they have to the current antibacterial and antifungal agents [31]. This inhibitory activity may indicate a possible breakthrough in the fight against the challenges caused by drug resistant microorganisms such as reduced efficacy of drugs, costly or impossible treatment and increased mortality [32].

This study examined *B. macowanii*’s mechanism of action in order to assess its potential for use in evidence-based antimicrobial therapy. In the absence of any previous studies regarding the plant’s mechanism of action, electron microscopy was used to determine the effect of *B. macowanii* on bacterial cells. The morphological effects of *B. macowanii* on the bacterial cell wall is comparable to that of Vancomycin used to treat infections caused by *Bacillus cereus*. Vancomycin inhibits the second stage of the cell wall synthesis, impairs the cell membrane permeability and inhibits ribonucleic acid synthesis [33]. The minimum inhibitory concentration of the plant extract on *B. cereus* was 1.2 mg/ml using the broth microdilution method; however, under microscopy, cells treated with the plant extract at lower concentrations also showed some significant morphological changes. According to our electron microscopy findings, bacterial cells were affected morphologically at concentrations ranging from 0.125–2.5 mg/ml. A recent study showed that *Melastoma candidum* through SEM also had morphological effects on the cells of *E. coli* at a concentration of (0.6–5.0 mg/ml) [34]. In contrast, pure compounds such as chlorogenic acid, curcumin, epicatechin, eugenol, myricetin, quercetin, rutin, thymol, thymoquinone, and xanthohumol affect the cell morphology of *Escherichia coli* and *Salmonella cholerasuis at* much lower concentrations (0.01 mg/ml). Further investigation into the mechanism of action of isolated pure compounds from *B. macowanii* may demonstrate similar effects on cell morphology and indicate potential for the development of alternative antimicrobial agents [35].

Neophytadiene is also used for treatment of headaches, rheumatism and some skin diseases [36]. Neophytadiene is considered to have antimicrobial properties [37, 38] and may account for the antimicrobial activity demonstrated in this study. Most plants from the Buxus genus such as *Buxus rugulose*, are known to contain triterpenoid alkaloids which have different biological activities, including antimicrobial activity [36]. Additionally, five new steroidal alkaloids: 31-hydroxybuxatrienone, macowanioxazine, 16a-hydroxymacowanitriene, macowanitriene and macowamine as well as five known steroidal bases Nb-demethylpapillotrienine, moenjodaramine, irehine, buxbodine B and buxmicrophylline C have recently been isolated from *B. macowanii* [39]. Steroidal alkaloids are also known to cause cell membrane disruption and consequently, leakage of cytoplasmic contents [40]. This couldexplain the disruption of the cell membrane and the loss of cytoplasmic contents (Fig. 1b and c) observed on bacterial cells treated with *B. macowanii.* Furthermore, the steroidal alkaloids found in the plant have anti-AChE activity or showed prevention of excessive degradation of acetylcholine which is associated with Alzheimer’s disease [39], indicating a multipurpose remedy.

Although alkaloids are associated with the above-mentioned biological properties, it has been reported that alkaloids may be toxic to humans and animals; caution must therefore be taken in the consumption [41]. Cytotoxicity evaluation showed methanol extract of the leaves and twigs of *B. macowanii* as a moderate hazard against human fetal lung fibroblast and showed little selectivity for microbial cells. This may be of concern since a lack of selectivity reduces the therapeutic potential of plant extracts. However, selectivity may be improved, and toxicity reduced upon the isolation of the antibacterial compounds [27].

## Conclusion

*B. macowanii* showed antimicrobial activity against bacterial species *S. aureus, C. perfringens, P. aeruginosa, E. faecalis, E. coli, S. epidermidis, B. cereus* and the fungal species *C. albicans* and *C. tropicalis*. *B. macowanii* also caused morphological damage to the cell wall of *B. cereus.* GCMS analysis of the plant extract also detected neophytadiene which may contribute to the antimicrobial activity. This study constitutes the first report on the antimicrobial activity and the effect of *B. macowanii* on the cell wall of bacteria. Further investigations on isolated compounds from *B. macowanii* are recommended.

## Data Availability

All the data obtained, and materials analysed in this research are available from the corresponding author upon request.

## Abbreviations

GCMS: Gas Chromatography Mass Spectrometry
HIV: Human Immunodeficiency Virus
AIDS: Acquired Immune Deficiency Syndrome
ATCC: American Type Culture Collection
MIC: Minimum Inhibitory Concentration
SEM: Scanning Electron Microscopy
TEM: Transmission Electron Microscopy
TIC: Total Ion Chromatograms
AMDIS: Automated Mass Spectral Deconvolution and Identification Software
NIST: National Institution of Standard Technology
AChE: Acetylcholinesterase
DHET: Department of Higher Education and Training
CUT: Central University of Technology
UFS: University of Free State
SRB: Sulforhodamine B
ECACC: European Collection of Cell Culture
EMEM: Eagle’s Minimum Essential Medium
FBS: Fetal Bovine Serum
SI: Selectivity Index
